# The Metalloproteinase *adam19b* Is Required for Sensory Axon Guidance in the Hindbrain

**DOI:** 10.3389/fncir.2019.00014

**Published:** 2019-03-06

**Authors:** Jane A. Cox, Mark M. Voigt

**Affiliations:** Department of Pharmacology and Physiology, Saint Louis University School of Medicine, St. Louis, MO, United States

**Keywords:** axon guidance, sensory circuits, zebrafish, metalloprotease, cranial nerves

## Abstract

Little is known about the molecular and cellular mechanisms involved in the formation of the cranial peripheral sensory system in vertebrates. To identify genes involved in the formation of these circuits, we performed a forward genetic screen utilizing a transgenic zebrafish line (*p2rx3.2:gfp*^sl1^) that expresses green fluorescent protein (gfp) in sensory neurons of the Vth, VIIth, IXth and Xth cranial ganglia. Here, we describe a novel zebrafish mutant in which a missense mutation in the *adam19b* gene selectively affects the epibranchial sensory circuits.

## Introduction

The peripheral sensory nervous system serves as an interface between the internal and external environments and the central nervous system (CNS). The information it carries must be relayed with precision so that appropriate behavioral and/or physiological responses are made. A subset of cranial nerves (V, VII, IX and X) containing both motor and sensory axons innervates structures in the head and neck, as well as the non-pelvic viscera. The sensory axons within these nerves originate from four distinct cranial sensory ganglia (CSG): the trigeminal (Vth), the facial (VIIth), the glossopharyngeal (IXth) and the vagal (Xth). These neurons transduce somatosensory, chemosensory and viscerosensory stimuli from peripheral receptors/organs and terminate within discrete regions of the hindbrain in a topographic fashion (Butler and Hodos, 2005). However, the genetic programs responsible for establishment of these circuits are poorly understood.

A powerful approach to identify genes involved in developmental programs is the forward genetic screen. To identify genes utilized in sensory circuit formation, we carried out a forward screen in a transgenic zebrafish line Tg(*p2rx3.2:gfp^sl1^*; *3.2:gfp*) that expresses green fluorescent protein (gfp) in sensory neurons of the Vth, VIIth, IXth and Xth cranial ganglia (Kucenas et al., 2006). One mutant line, *3.2:gfp*^sl19^ (*sl19*), showed aberrant projections of only the epibranchial axons. Here, we present data showing *sl19* is the metalloprotease *adam19b* and is critical for sensory circuit formation.

## Materials and Methods

### Maintenance of Fish

All animal husbandry and staging was carried out according to Kimmel et al. (1995). Embryos used for microscopy were treated with 0.003% phenylthiourea to reduce pigmentation (for transgenic fish lines used in this study, see Supplementary Table S1).

### Positional Cloning

Standard mapping methods using bulk segregant and meiotic recombination analysis of microsatellite markers (Green et al., 2009; Cox et al., 2014) were used to map the *sl19* mutation to a critical interval on chromosome 14. Total RNA was then extracted from mutant and wild type 4 days post fertilization (dpf) larvae and cDNA synthesized with Superscript III reverse transcriptase (Invitrogen, Carlsbad, CA, USA). cDNAs encoding candidate genes within the critical interval were sequenced using gene specific primers.

### Embryo Injections

RNA (100 pg/nl) or morpholino oligonucleotides (MO; Gene Tools, 1–2 ng/nl, Supplementary Table S2) were injected as previously described (Cox et al., 2014).

### Imaging of Larvae

Imaging was carried out as described previously (Cox et al., 2014). Final brightness and/or contrast values of images were adjusted in Adobe Photoshop CC 2017.

### Immunohistochemistry

This was performed as described in a previous article (Cox et al., 2014). Images were obtained by confocal microscopy as described above.

### Wholemount *in situ* Hybridization

*In situ* hybridization (ISH) was performed as previously described (Kucenas et al., 2006). Primers were designed from the predicted sequence of the zebrafish *adam19b* mRNA (Supplementary Table S3) and a 600 bp fragment was obtained by PCR from 4-day-old zebrafish cDNA. This fragment was cloned into pBSII(+; Promega) and used to prepare digoxigenin-labeled RNA.

### CRISPR/Cas9 Gene Editing

An RNA oligo complementary to a sequence in exon 2 of *adam19b* was designed using http://chopchop.cbu.uib.no/ (Supplementary Table S4). Alt-R oligos were synthesized by IDT. A complex consisting of Alt-R oligos and Cas9 protein (IDT) was injected into single cell zebrafish embryos according to the method of Essner as reported on the IDT website. To validate CRISPR/Cas9 targeting, genomic DNA was isolated at 4 dpf from control and injected larvae, exon 2 was amplified by PCR (see Supplementary Table S4 for primers) and the products sequenced.

## Results

### The *sl19* Mutation Selectively Affects Cranial Sensory Circuits

**Figure 1:**
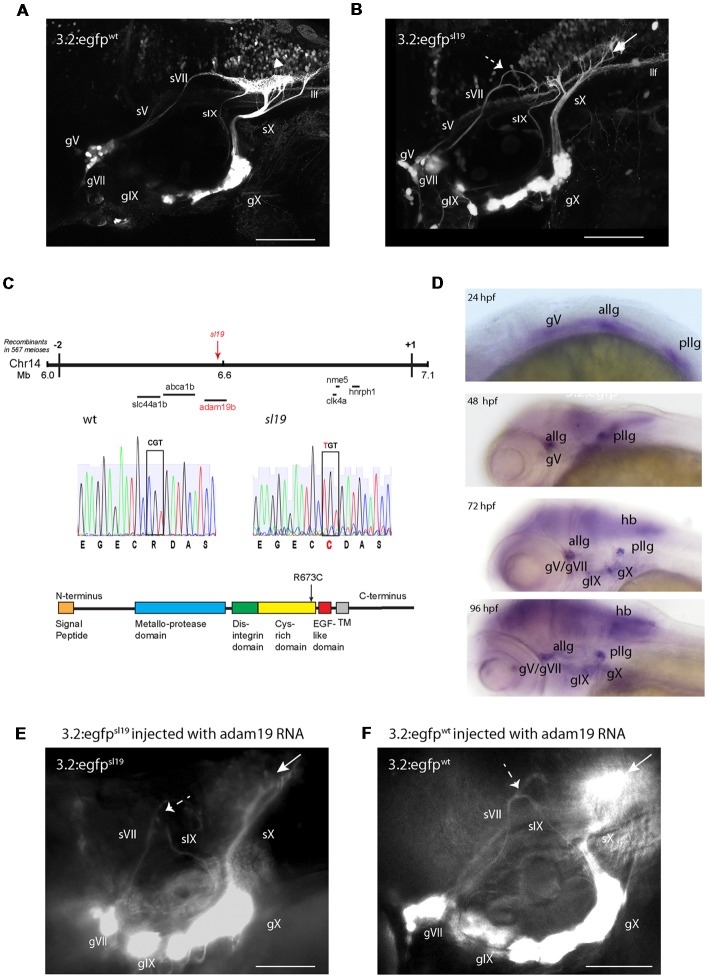
Characterization of the *sl19* mutant line. **(A)** Wild type larva at 4 days post fertilization (4 dpf) showing normal pattern of cranial sensory ganglia (CSG) projections. sV (also llf-lateral longitudinal fascicle), sVII, sIX and sX are the projections of the trigeminal (gV), facial (VII), glossopharyngeal (IX) and vagus (X) ganglia, respectively. White arrowhead shows normal hindbrain plexus, where sVII, sIX and sX sensory axons terminate. **(B)** CSG projections in a 4dpf *sl19* mutant. White arrow indicates malformed hindbrain plexus and dotted arrow shows the misrouted and defasciculated sVII failing to terminate in the plexus. sIX and sX fail to form normal terminal fields. Projections from gV appear normal. **(C)** Positional cloning of *sl19*. The lesion was mapped to a critical region on chromosome 14 between 6 and 7 Mb. Transcripts of all genes in this region (20 in total) were sequenced (for clarity, only some of these genes are shown in the Figure). Only the *adam19b* gene showed an altered coding sequence—a single base mutation in exon 17. This C > T conversion causes an arginine to cysteine switch at amino acid 673 in the cysteine-rich domain (CRD) of A Disintegrin and Metalloproteinase (*ADAM*) *19b* (CRD). **(D)** Wholemount *in situ* hybridization (ISH) of *adam19b* in the zebrafish head. At 24 hpf, *adam19b* is expressed in the trigeminal ganglion (gV), the anterior lateral line ganglion (allg) and the posterior lateral line ganglion (pllg). At 48 hpf, expression has expanded to include regions of the hindbrain. By 72 hpf, *adam19b* is also expressed in the epibranchial ganglia (gVII, gIX and gX) and in the hindbrain (hb); by 96 hpf, expression has increased in all these cranial structures. Sense probe did not produce staining at any time point (data not shown). **(E)** *adam19b* full length RNA injected into *sl19* mutant. Over-expression of *adam19b* did not rescue the mutant phenotype, but resulted in a higher incidence of the mutant phenotype (see text). White arrow indicates malformed plexus; dotted arrow shows misrouted sVII and sIX. **(F)** *adam19b* full length RNA injected into wild type. As can be seen, over-expression of *adam19b* induced aberrant epibranchial projections in 4 dpf wild type fish, identical to that found in the *sl19* mutant. White arrow: malformed plexus, showing how sX forms a dense clump of axons, instead of the organized branching of the axons seen in un-injected controls **(A)**. Dotted arrow: misrouted sVII and sIX. Panels **(A,B)** are confocal z-stacks, panels **(E,F)** are epifluorescent micrographs. All images are oriented anterior to the left and dorsal at top. Scale bars = 100 μm.

A forward genetic screen investigating zebrafish cranial sensory circuit formation was performed using the reporter strain *3.2:gfp* (Kucenas et al., 2006; Figure 1A, Supplementary Figures S1). *3.2:gfp*^sl19^ (*sl19*) was one of several mutant lines detected. *sl19* showed a recessive Mendelian inheritance: mutants constituted 27% ± 6 of larvae in a clutch (*n* = 235/870 from nine clutches). By 4dpf, the *sl19* line contained defects in the central projections of the epibranchial ganglia (gVII, gIX and gX; Figure 1B, Supplementary Figures S1); in all cases the axons projected to the hindbrain as normal, but once in the hindbrain, the axons showed aberrant pathfinding to their targets. While there was considerable variability in the misrouting of the axons, gVII axons typically failed to turn caudally towards the afferent plexus in the viscerosensory column and instead continued in a dorsal direction with no discernable terminal field (Supplementary Figures S1D,F). In some mutants, gIX axons turn rostrally instead of caudally towards the plexus and stop anteriorly to gVII nerve (Supplementary Figures S1C,F); sometimes gIX axons pathfind rostrally, then join gVII and turn towards the plexus Supplementary Figure S1E. In most cases, gX axons do not branch to form a normal hindbrain plexus (Supplementary Figures S1C,D,F). The gV projections do not appear to be affected in this mutant (Figure 1B, Supplementary Figure S1). These phenotypes were never observed in wild-type larvae (*n* > 5,000).

### The *sl19* Mutation Maps to the Metalloprotease Gene *adam19b*

Linkage mapping indicated that the *sl19* allele was located on Chromosome 14, and high-resolution positional cloning identified a C > T mutation located within exon 17 of *adam19b* (Figure 1C) as the potential causative lesion. This nucleotide switch converts an arginine to a cysteine at amino acid 673. *adam19b* encodes a member of the A Disintegrin and Metalloproteinase (ADAM) family, and is a 923 amino acid Type I transmembrane protein composed of a series of conserved domains that include: a signal sequence, a pro-domain, a metalloproteinase domain, a disintegrin domain, a cysteine-rich domain (CRD), an EGF-like domain, a transmembrane region and a C-terminus containing signaling-related motifs (e.g., SH3 sequences; White, 2003; Figure 1C). The *sl19* mutation is within the CRD of the protein, a region that has been shown to be critical for the protein’s metalloprotease activity (Smith et al., 2002; Kang et al., 2004). Supplementary Figure S2 shows an alignment of several vertebrate CRDs.

### *adam19b* Expression Includes the Cranial Sensory Ganglia and Hindbrain

Whole mount ISH revealed *adam19b* mRNA in the CSG (Figure 1D). Expression was detectable in the ganglia early, present in the trigeminal (gV) and both lateral line ganglia at 24 hpf. In 48 hpf embryos, expression was detected in the CSG and also in CNS structures such as the cerebellar plate and optic tectum. By 72 hpf, *adam19b* expression included all of the CSG as well as cells in the retina, olfactory epithelium and hindbrain: this was also the pattern seen in 4 dpf larvae.

### Ectopic/Overexpression of *adam19b* RNA Induces a Phenotype Resembling *sl19*

One approach to validating the identity of a candidate gene is to rescue the phenotype of the mutant by injection of the wild type full-length RNA. When full length *adam19b* RNA was injected into embryos from *sl19*^+/–^ in-crosses (three separate experiments), we unexpectedly observed a significant increase in the number of 4 dpf larvae exhibiting the mutant phenotype: 47% (57/121) of injected embryos had a mutant phenotype compared to 25% in un-injected clutchmates (*n* = 26/102; *p* < 0.0013, Fisher’s exact test; Figure 1E). This suggested that over-expression of *adam19b* induced perturbations in afferent circuits. To validate this hypothesis, full length *adam19b* RNA was injected into single-cell wild type *3.2:GFP* embryos. This resulted in disruption of the epibranchial afferents, phenocopying *sl19*, in 24% of the 4 dpf embryos (9/37; Figure 1F). These findings indicate that appropriate guidance molecule processing by *adam19b* is critical for epibranchial axon pathfinding.

### Knockdown of *adam19b* in Wild-Type Embryos Phenocopies *sl19*

**Figure 2:**
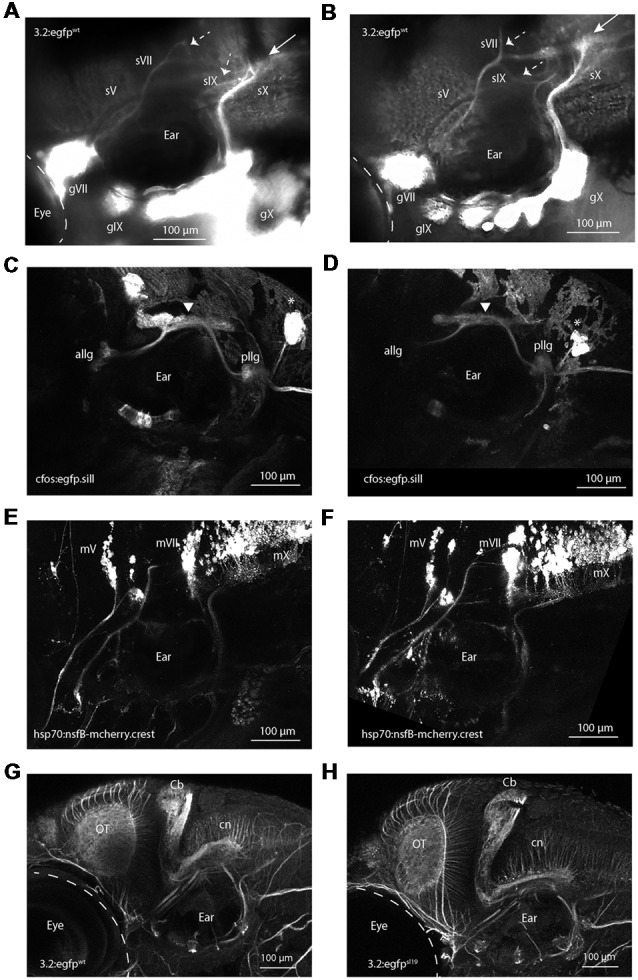
Validation of *adam19b* as critical for epibranchial sensory axon pathfinding. **(A)** *adam19b* start morpholino (MO1; 4 ng) injected into *Tg(p2rx3.2:gfp)* embryos. This image shows the disruption of the projections of sVII and sIX (dotted arrows) and the malformed plexus (white arrow), recapitulating the phenotype of *sl19*. sV appears unaffected. A 5 bp-mismatched MO gave no phenotype (for image of control fish, see Figure 1A).** (B)** Phenotype from CRISPR targeting of *adam19b* exon 2. White arrow shows malformed plexus and dotted arrows show disrupted axon projections of the sVII and sIX, again phenocopying *sl19* (for image of control fish, see Figure 1A). Injection of gRNAs targeting other genes (e.g., *drg11, golden*) did not produce these phenotypes). sVII, sIX and sX are the projections of the facial (gVII), glossopharyngeal (gIX) and vagus (gX) ganglia, respectively.** (C)** Confocal image of 4 dpf *Tg(3.2:nsfB-mcherry/cfos:gfp.sill)* wild-type larvae, showing the anterior lateral line ganglia (allg) and the posterior lateral line ganglia [pllg; green fluorescent protein (gfp) channel: *cfos:gfp.sill*), which project to the lateral line plexus (white arrowhead) in the hindbrain. **(D)** Confocal image of 4 dpf *Tg(3.2:nsfB-mcherry/cfos:gfp.sill*] *adam19b* CRISPR/Cas9 mutants demonstrating the lateral line projections to the hindbrain are not affected by the *adam19b* CRISPR indels (gfp channel: *cfos:gfp.sill*) *neuromasts. **(E)** Confocal image of banchiomotor nerves in 4 dpf wild-type *Tg(3.2:gfp/hsp70:nsfB-mcherry.crest)* larvae, showing the segmental organization of the motor neurons (mV, mVII and mX; red channel: *hsp70:nsfB-mcherry.crest*). **(F)** Confocal image of branchiomotor nerves in *Tg(3.2:gfp/hsp70:nsfB-mcherry.crest). adam19b* CRISPR/Cas9 mutants show that the segmental organization of the motor nuclei (mV, mVII and mX) and their projections appear normal (red channel: *hsp70:nsfB-mcherry.crest*). **(G,H)** The hindbrain organization is not affected in *sl19* mutants. Anti-acetylated tubulin immunostaining of 4 dpf wild-type **(G)** and *sl19* mutant larvae **(H)** shows no disruption to the cerebellum (Cb), the optic tectum (ot) or the commissural neurons (cn) in the mutants. For panels **(C–F)**, cognate images in the channel showing epibranchial afferents are shown in Supplementary Figures S5, S6. All images are oriented anterior to the left and dorsal at top. Scale bars = 100 μm.

We predicted that knock-down of *adam19b* would phenocopy the *sl19* mutant. To test this, we used a morpholino targeting the start codon of *adam19b* (MO1). Injection of MO1 (2–4 ng) resulted in 19% of injected 4 dpf larvae exhibiting a phenotype resembling *sl19*: misrouting of the projections of the VIIth, IXth and Xth ganglia, but not those of the Vth (Figure 2A; *n* = 29 out of 156, six clutches vs. 0/127 of un-injected controls, *p* < 0.0001, Fisher’s Exact Test). Injection of a second morpholino (MO2), designed against the e17i17 splice junction, gave similar phenotypes (Supplementary Figure S3). To further validate *adam19b* as the affected gene and to address known concerns with the use of morpholinos, CRISPR/Cas9 targeting of exon 2 in *adam19b* in wild-type *3.2:GFP* embryos was performed. These 4 dpf larvae phenocopied *sl19* (*n* = 11 out of 49, three clutches, vs. 0/54 un-injected controls, *p* < 0.0001 Fisher’s Exact Test; Figure 2B). Confirmation of CRISPR/Cas9 targeting by sequencing is shown in Supplementary Figure S4. Combined, these results strongly support a role for *adam19b* in epibranchial afferent pathfinding.

### Knockdown of *sl19* Has No Effect on the Non-epibranchial Cranial Nerves

To determine if *adam19b* is required for the formation of other peripheral cranial circuits, we again turned to CRISPR/Cas9 targeting of exon 2 in transgenic fish. In 4 dpf larvae exhibiting defects in epibranchial circuits (Supplementary Figures S5, S6), we did not detect any perturbations in either lateral line circuits [Tg*(p2rx3.2GR:nsfB-mcherry;cfos:gfp.sill)*; Figures 2C,D; Supplementary Figure S5; *n* = 0/41 or branchiomotor projections (Tg(*p2rx3.2:gfp;hsp70:nsfB-mcherry.crest*)); Figures 2E,F; Supplementary Figure S6; *n* = 0/32]. These results suggest that *adam19b* is utilized selectively by the epibranchial sensory neurons in their pathfinding to hindbrain targets.

### *sl19* Does Not Affect Hindbrain Organization

The configuration of the branchiomotor neurons and their axons was not affected at 4 dpf by *adam19b* CRISPR/Cas9 targeting (Figures 2E,F). This suggests that *adam19b* does not have a critical role in the segmental organization of the hindbrain. To verify this, we used immunostaining of acetylated tubulin, which labels axons throughout the CNS and periphery. As seen in Figures 2G,H, there are no gross perturbations in the CNS in *sl19* mutants at 4 dpf (*n* = 52): the organization of the optic tectum, the cerebellum and commissural neurons appear normal.

## Discussion

ADAMs are transmembrane zinc metalloproteases that contain multiple functional domains (Seals and Courtneidge, 2003). Members of this family have been shown to be involved in numerous cellular processes, such as adhesion, migration, proteolysis and signaling (Wolfsberg et al., 1995; Primakoff and Myles, 2000; Seals and Courtneidge, 2003; White, 2003). One member of this family, *adam19*, has been reported to have a role in neural crest migration, neuromuscular junction formation and cardiac development (Kurohara et al., 2004; Yumoto et al., 2008; Neuner et al., 2009; Schiffmacher et al., 2014). *Adam19* is expressed in many tissues including the CSG (Kurisaki et al., 1998; Shirakabe et al., 2001; Yan et al., 2011), but its function in these neurons is unknown. In this report we demonstrate that the zebrafish ortholog, *adam19b*, plays a critical role in epibranchial sensory circuit formation.

We have identified a mutant, *sl19*, in which only the epibranchial afferents are misrouted. Positional cloning revealed a lesion (R673C) in the CRD of *adam19b*. Studies have shown that this region is critical for the post translational processing necessary for its protease and disintegrin functions (Blobel, 1997; Smith et al., 2002). Kang et al. (2002, 2004) have shown that the disulfide bonds formed between the cysteine residues in the CRD are critical for sheddase activity in the human *ADAM19*. We hypothesize that in *sl19* the introduction of an extra cysteine leads to disordered formation of intra-domain disulfide bonds, disrupting the topology of the CRD and resulting in abnormal folding of the protein. As a result, *adam19b* could fail to translocate to the cell surface, or alternatively the CRD may not fold properly, resulting in either lack of conversion to an active form or alteration in the substrate specificity of the active form. Since the phenotype is recapitulated by depletion of *adam19b* in wild type larvae, we favor the hypothesis that *sl19* is a loss of function mutant, rather than one with altered function; such a loss would result in disruption of the guidance cue recognition by these axons, leading to their variable misrouting. Given the expression of *adam19b*, it remains to be determined whether it is loss of function in axons or in the hindbrain (or both) that is responsible for the defects found.

It is striking that only the epibranchial sensory axons are affected in zebrafish in *sl19* and not other circuits such as the trigeminal, lateral line or branchiomotor nerves. This suggests that the molecular targets of *adam19b* are critical for axon guidance of viscerosensory afferents, but not mechanosensory or branchiomotor axons. The identification of these substrates will require additional investigation.

## Data Availability

All datasets generated for this study are included in the manuscript and/or the supplementary files.

## Ethics Statement

All experiments were carried out in accordance with the National Institutes of Health Guide for the Care and Use of Laboratory Animals. The Saint Louis University Institutional Animal Use and Care Committee approved the procedures used (protocol #2647).

## Author Contributions

Both authors contributed to the conception and design of the study, to manuscript revision, read and approved the submitted version.

## Conflict of Interest Statement

The authors declare that the research was conducted in the absence of any commercial or financial relationships that could be construed as a potential conflict of interest.

## Supplementary Material

FIGURE S1 Variations in *sl19* phenotype in Tg(*p2rx3.2:gfp*) embryos. sV (also labeled as llf), sVII, sIX and sX are the projections of the trigeminal (gV), facial (gVII), glossopharyngeal (gIX) and vagus (gX) ganglia, respectively. Panel **(A)** shows wild type *p2rx3.2:gfp*. Panel **(B)** is a schematic showing the ganglia and their projections in matching colors. Panels **(C–F)** are images from individual *sl19* mutants. Panel **(C)** shows sVII defasciculating, with the majority of fibers stopping before reaching the plexus and the few fibers that do reach the plexus do so in an aberrant fashion. sIX joins sX before it enters the plexus, and sX shows aberrant branching. In **(D)**, the main bundle of sIX stops in the hindbrain while a small number of fibers make a caudal turn towards the plexus. sX also shows a small degree of defasciculation by individual axons. Panel **(E)**, sVII defasiculates and sIX continues in an anterior direction towards sVII. sX fails to branch properly but does contribute to the plexus. Panel **(F)**, sVII again defasciculates and fails to reach the region of the plexus, sIX stops in the hindbrain and fails to form a terminal field, and sX shows abnormal branching and also fails to form a normal terminal field. Panel **(G)** is a schematic showing a selection of the aberrant phenotypes seen in *sl19* mutants. All images are confocal z-stacks oriented anterior to the left and dorsal at top. Scale bars = 100 μm.

FIGURE S2 Partial amino acid sequence alignment of *adam19* in rat, mouse, human, zebrafish and *sl19* (aa 481–720). The underlined area is the Cysteine Rich Domain. The amino acids highlighted in yellow are conserved across all species. The conserved cysteine residues are highlighted in red. The arginine residue in blue is conserved in all species, but in *sl19* is mutated to a cysteine (marked by a red asterisk).

FIGURE S3 A second morpholino (MO2) was designed against the e17i17 splice junction resulting in an in-frame deletion of exon 17 (corresponding to aa 666–691), leading to disruption of the CRD (**A**; sequence shown in Supplementary Table S2). Injection of MO2 (4–8 ng/nL) into *3.2:gfp* embryos resulted in mis-routing of the epibranchial projections, similar to MO1. sVII, sIX and sX are the projections of the facial (gVII), glossopharyngeal (gIX) and vagus (gX) ganglia, respectively. Note the disruption of sX afferents and lack of hindbrain plexus. All images are oriented anterior to the left and dorsal at top. Scale bars = 100 μm. **(B)** Verification of effectiveness of MO2. cDNA was synthesized from four 48 hpf embryos injected with MO2 and a control, uninjected 48 hpf embryo. Primers E16.F and E18.R were designed (see Supplementary Table S2) to amplify a 280 bp fragment in control embryos (last lane on right, **). In MO2 injected embryos (lanes 1–4), a 200 bp fragment (*) was amplified, indicating a loss of exon 17 (80 bp). There is a non-specific PCR artifact at 150 bp (†), which appears in all reactions.

FIGURE S4 Verification of CRISPR/cas9 targeting of exon 2. Genomic DNA was isolated from four dpf larvae that were injected with CRISPR/cas9+gRNA and showed defective epibranchial axon guidance. Exon 2 (which contains the targeted sequence) was amplified using PCR and sequenced. Results from four mutant larvae demonstrated that multiple indel-containing sequences were present (three examples from each larva are shown). These results are consistent with mosaic biallelic targeting by CRISPR/cas9 in F0 individuals. Un-injected larvae yielded amplicons only corresponding to the wild-type genomic sequence (*adam19b* reference).

FIGURE S5 Crispr/Cas9 targeting of *adam19b* in Tg(*3.2:nsfB-mcherry:cfos:gfp.sill*) larvae expressing egfp in lateral line ganglia and *mCherry* in epibranchial sensory ganglia. Panels **(A–C)** represent control fish: **(A)** shows lateral line ganglia (green channel:*cfos:gfp.sill*). allg, anterior lateral line ganglia; pllg, posterior lateral line ganglia. Panel **(B)** shows epibranchial ganglia (red channel:*3.2:nsfB-mcherry*; sVII, sIX and sX are the projections of the facial (gVII), glossopharyngeal (gIX) and vagus (gX) ganglia, respectively) and **(C)** shows a composite of both images. Panels **(D–F)** represent Crispr/Cas9 targeting of *adam19b*. Panel **(D)** shows lateral line ganglia with unaffected projections (green channel::*cfos:gfp.sill*), **(E)** shows misrouted epibranchial axons (dotted white arrow; red channel:*3.2:nsfB-mcherry*) and **(F)** shows a composite of both images. All images are oriented anterior to the left and dorsal at top. Scale bars = 100 μm.

FIGURE S6 Crispr/Cas9 targeting of *adam19b* in Tg(*3.2:egfp:hsp70:nsfB-mcherry.crest*) larvae expressing *mCherry* in motor neurons and egfp in epibranchial sensory ganglia. Panels **(A–C)** represent control fish: **(A)** shows motor neurons and axons (mV, mVII and mX; red channel:*hsp70:nsfB-mcherry.crest*), **(B)** shows epibranchial sensory ganglia (green channel:*3.2:egfp*; sVII, sIX and sX are the projections of the facial (gVII), glossopharyngeal (gIX) and vagus (gX) ganglia, respectively) and **(C)** shows a composite of both images. Panels **(D–F)** represent Crispr/Cas9 targeting of *adam19b*. Panel **(D)** shows unaffected mV, mVII and mX motor neurons (red channel:*hsp70:nsfB-mcherry.crest*), **(E)** shows misrouted epibranchial ganglia projections (dotted white arrow; green channel:*3.2:egfp*) and **(F)** shows a composite of both images. All images are oriented anterior to the left and dorsal at top. Scale bars = 100 μm.

TABLE S1 Transgenic zebrafish lines used in this study.

TABLE S2 Morpholino oligonucleotides.

TABLE S3 Primers used to produce a 600 bp PCR fragment for whole mount *in situ* hybridization.

TABLE S4 Primers for Crispr/Cas9 targeting and PCR.
