# Supplementary figures and images for: The Metalloproteinase adam19b Is Required for Sensory Axon Guidance in the Hindbrain
Source: Front Neural Circuits. 2019 Mar 6;13:14. doi: 10.3389/fncir.2019.00014 (PMC6415755; doi:10.3389/fncir.2019.00014)

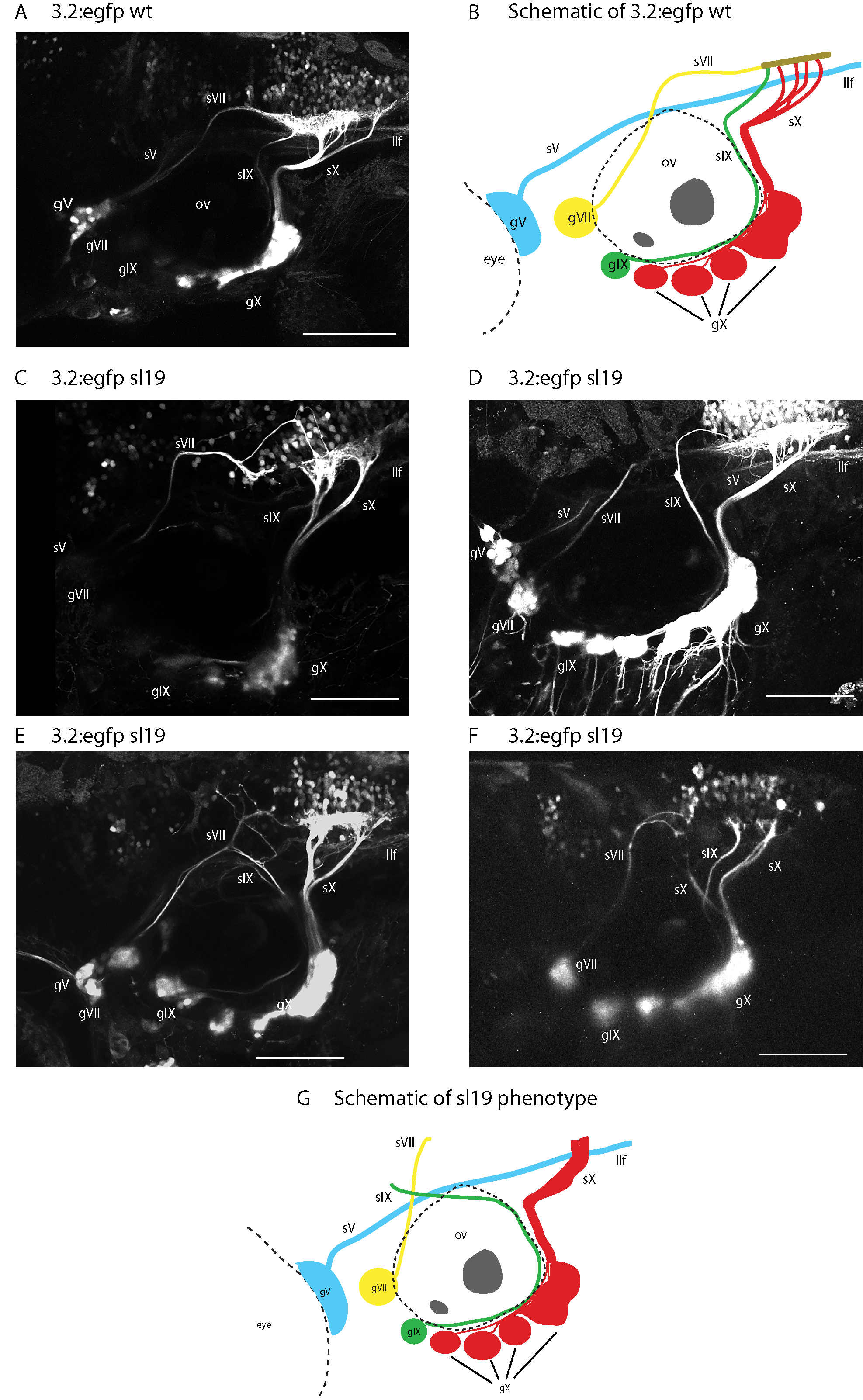

Supplement: FIGURE S1 — Variations in sl19 phenotype in Tg(p2rx3.2:gfp) embryos. sV (also labeled as llf), sVII, sIX and sX are the projections of the trigeminal (gV), facial (gVII), glossopharyngeal (gIX) and vagus (gX) ganglia, respectively. Panel (A) shows wild type p2rx3.2:gfp. Panel (B) is a schematic showing the ganglia and their projections in matching colors. Panels (C–F) are images from individual sl19 mutants. Panel (C) shows sVII defasciculating, with the majority of fibers stopping before reaching the plexus and the few fibers that do reach the plexus do so in an aberrant fashion. sIX joins sX before it enters the plexus, and sX shows aberrant branching. In (D), the main bundle of sIX stops in the hindbrain while a small number of fibers make a caudal turn towards the plexus. sX also shows a small degree of defasciculation by individual axons. Panel (E), sVII defasiculates and sIX continues in an anterior direction towards sVII. sX fails to branch properly but does contribute to the plexus. Panel (F), sVII again defasciculates and fails to reach the region of the plexus, sIX stops in the hindbrain and fails to form a terminal field, and sX shows abnormal branching and also fails to form a normal terminal field. Panel (G) is a schematic showing a selection of the aberrant phenotypes seen in sl19 mutants. All images are confocal z-stacks oriented anterior to the left and dorsal at top. Scale bars = 100 μm. [file Image_1.TIF]

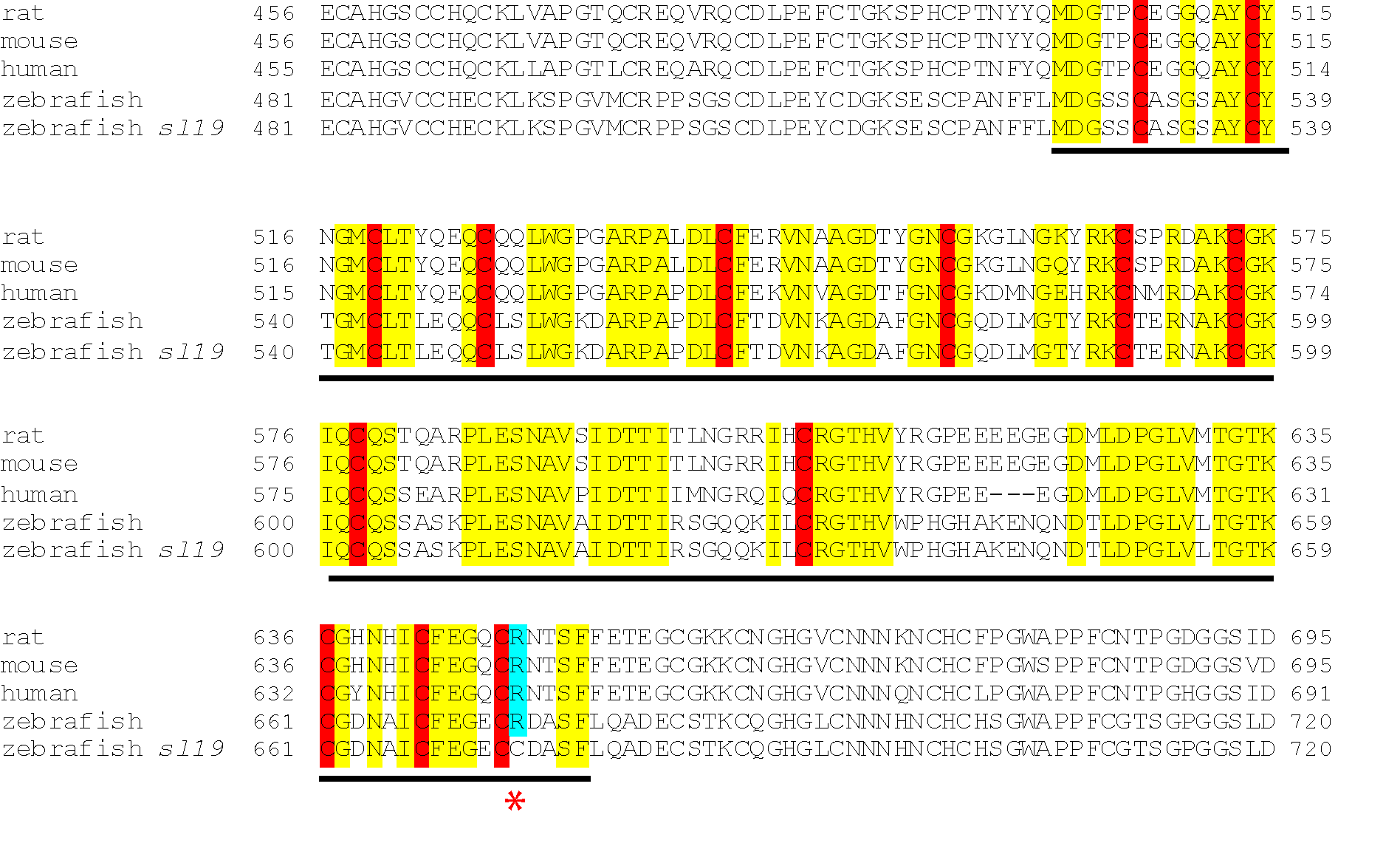

Supplement: FIGURE S2 — Partial amino acid sequence alignment of adam19 in rat, mouse, human, zebrafish and sl19 (aa 481–720). The underlined area is the Cysteine Rich Domain. The amino acids highlighted in yellow are conserved across all species. The conserved cysteine residues are highlighted in red. The arginine residue in blue is conserved in all species, but in sl19 is mutated to a cysteine (marked by a red asterisk). [file Image_2.TIF]

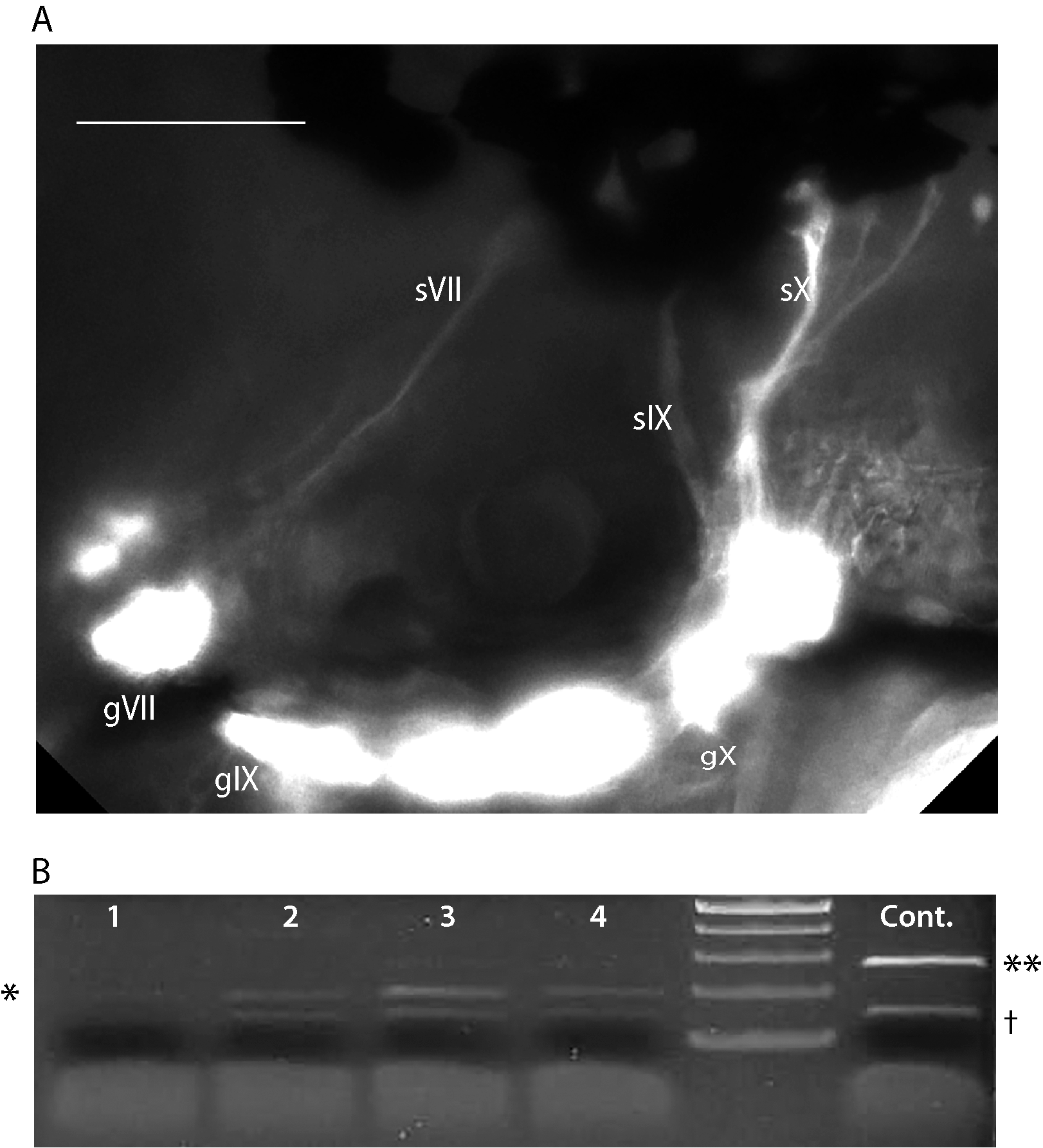

Supplement: FIGURE S3 — A second morpholino (MO2) was designed against the e17i17 splice junction resulting in an in-frame deletion of exon 17 (corresponding to aa 666–691), leading to disruption of the CRD (A; sequence shown in Supplementary Table S2). Injection of MO2 (4–8 ng/nL) into 3.2:gfp embryos resulted in mis-routing of the epibranchial projections, similar to MO1. sVII, sIX and sX are the projections of the facial (gVII), glossopharyngeal (gIX) and vagus (gX) ganglia, respectively. Note the disruption of sX afferents and lack of hindbrain plexus. All images are oriented anterior to the left and dorsal at top. Scale bars = 100 μm. (B) Verification of effectiveness of MO2. cDNA was synthesized from four 48 hpf embryos injected with MO2 and a control, uninjected 48 hpf embryo. Primers E16.F and E18.R were designed (see Supplementary Table S2) to amplify a 280 bp fragment in control embryos (last lane on right, **). In MO2 injected embryos (lanes 1–4), a 200 bp fragment (*) was amplified, indicating a loss of exon 17 (80 bp). There is a non-specific PCR artifact at 150 bp (†), which appears in all reactions. [file Image_3.TIF]

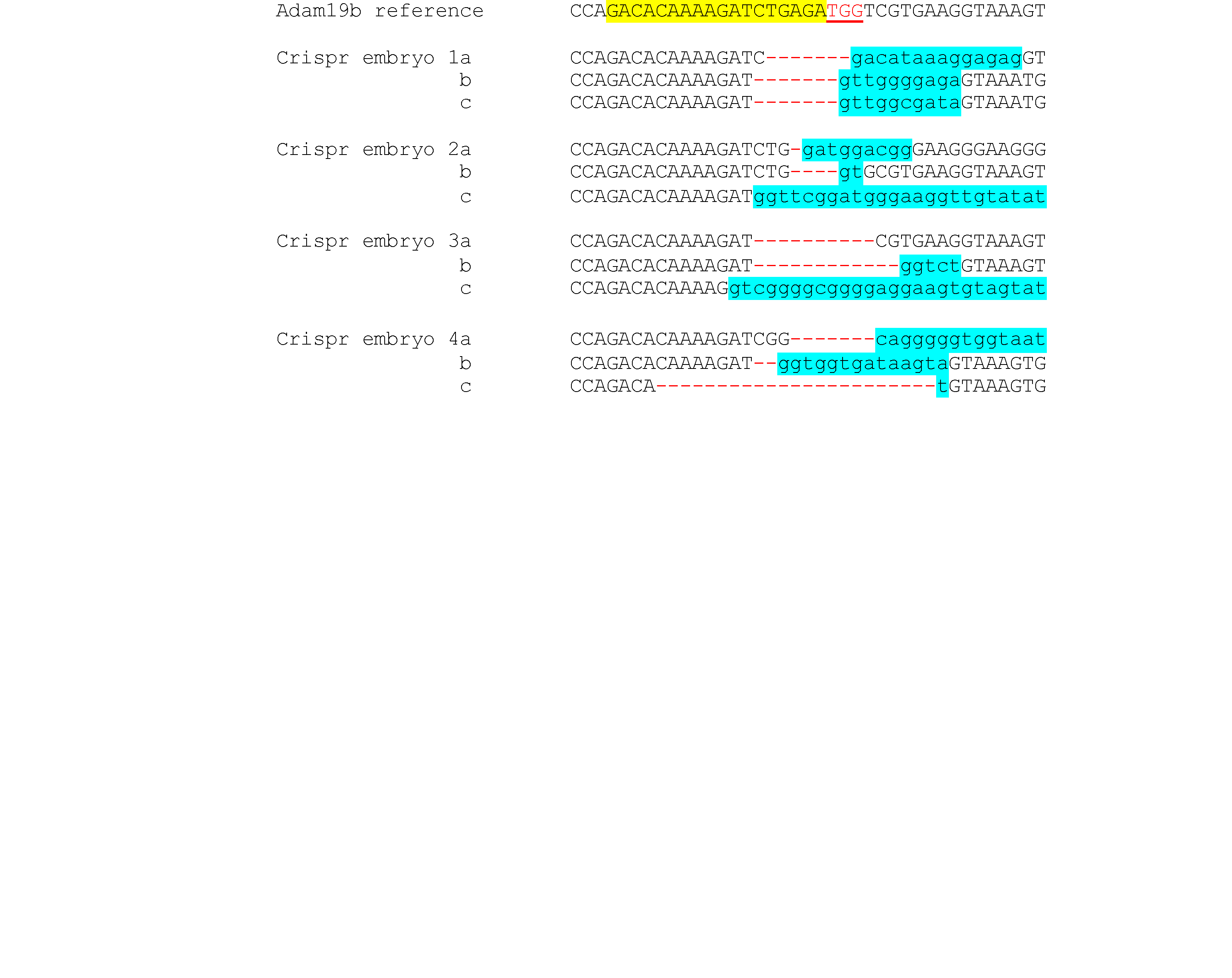

Supplement: FIGURE S4 — Verification of CRISPR/cas9 targeting of exon 2. Genomic DNA was isolated from four dpf larvae that were injected with CRISPR/cas9+gRNA and showed defective epibranchial axon guidance. Exon 2 (which contains the targeted sequence) was amplified using PCR and sequenced. Results from four mutant larvae demonstrated that multiple indel-containing sequences were present (three examples from each larva are shown). These results are consistent with mosaic biallelic targeting by CRISPR/cas9 in F0 individuals. Un-injected larvae yielded amplicons only corresponding to the wild-type genomic sequence (adam19b reference). [file Image_4.TIF]

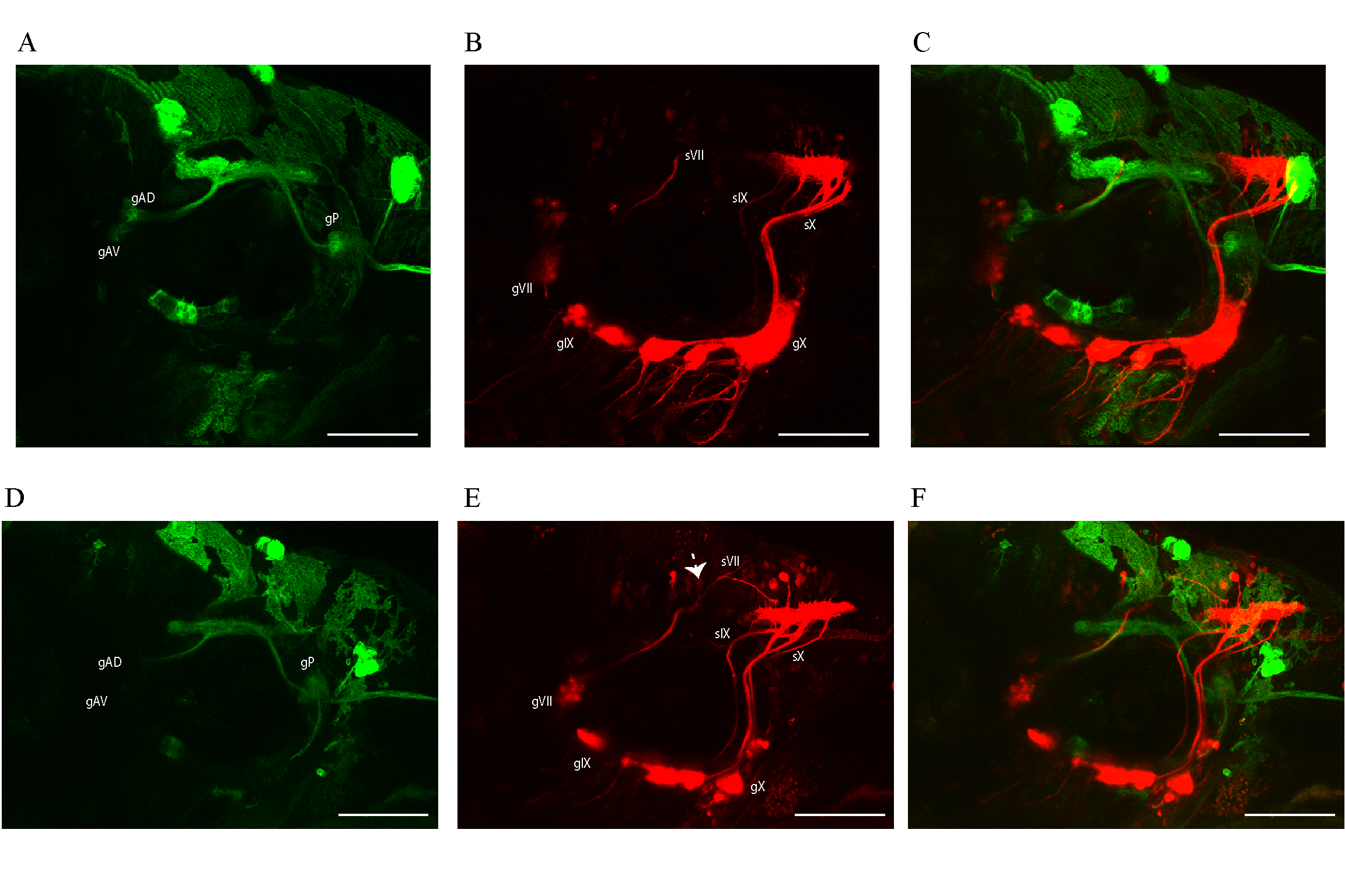

Supplement: FIGURE S5 — Crispr/Cas9 targeting of adam19b in Tg(3.2:nsfB-mcherry:cfos:gfp.sill) larvae expressing egfp in lateral line ganglia and mCherry in epibranchial sensory ganglia. Panels (A–C) represent control fish: (A) shows lateral line ganglia (green channel:cfos:gfp.sill). allg, anterior lateral line ganglia; pllg, posterior lateral line ganglia. Panel (B) shows epibranchial ganglia (red channel:3.2:nsfB-mcherry; sVII, sIX and sX are the projections of the facial (gVII), glossopharyngeal (gIX) and vagus (gX) ganglia, respectively) and (C) shows a composite of both images. Panels (D–F) represent Crispr/Cas9 targeting of adam19b. Panel (D) shows lateral line ganglia with unaffected projections (green channel::cfos:gfp.sill), (E) shows misrouted epibranchial axons (dotted white arrow; red channel:3.2:nsfB-mcherry) and (F) shows a composite of both images. All images are oriented anterior to the left and dorsal at top. Scale bars = 100 μm. [file Image_5.TIF]

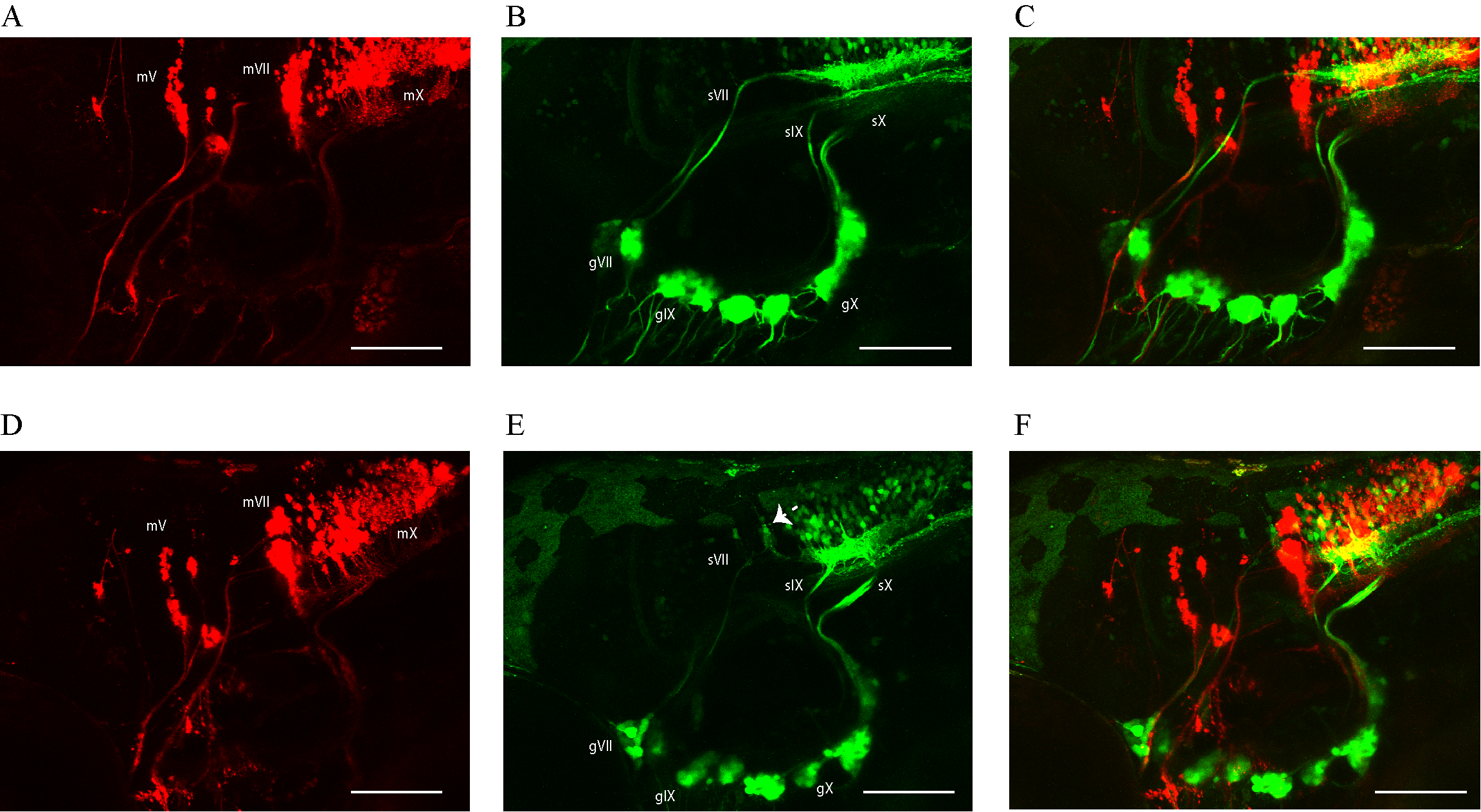

Supplement: FIGURE S6 — Crispr/Cas9 targeting of adam19b in Tg(3.2:egfp:hsp70:nsfB-mcherry.crest) larvae expressing mCherry in motor neurons and egfp in epibranchial sensory ganglia. Panels (A–C) represent control fish: (A) shows motor neurons and axons (mV, mVII and mX; red channel:hsp70:nsfB-mcherry.crest), (B) shows epibranchial sensory ganglia (green channel:3.2:egfp; sVII, sIX and sX are the projections of the facial (gVII), glossopharyngeal (gIX) and vagus (gX) ganglia, respectively) and (C) shows a composite of both images. Panels (D–F) represent Crispr/Cas9 targeting of adam19b. Panel (D) shows unaffected mV, mVII and mX motor neurons (red channel:hsp70:nsfB-mcherry.crest), (E) shows misrouted epibranchial ganglia projections (dotted white arrow; green channel:3.2:egfp) and (F) shows a composite of both images. All images are oriented anterior to the left and dorsal at top. Scale bars = 100 μm. [file Image_6.TIF]
