# Supplementary material for: The Metalloproteinase adam19b Is Required for Sensory Axon Guidance in the Hindbrain
Source: Front Neural Circuits. 2019 Mar 6;13:14. doi: 10.3389/fncir.2019.00014 (PMC6415755; doi:10.3389/fncir.2019.00014)
Supplement: TABLE S1 — Transgenic zebrafish lines used in this study. [file Table_1.docx]

Supplementary Table 1: Transgenic zebrafish lines used in this study

| Tg(*p2xr3.2:eGFP*) | Expresses eGFP in neurons of cranial sensory ganglia (gV, gVII, gIX, gX) (Kucenas et al. Neuroscience 138:641. 2006) |
| --- | --- |
| Tg*(p2rx3.2:nsfB-mcherry)* | Expresses bacterial nitroreductase in the gV, gVII, gIX and gX neurons |
| Tg*(cfos:egfp.sill)* | Expresses eGFP in the lateral line ganglionic neurons |
| Tg*(hsp70:nsfB-mcherry.crest)* | Expresses bacterial nitroreductase in the branchiomotor neurons (mV, mVII, mX) (Allen et al. J. Neurogenetics 31:128. 2017) |
