# Supplementary material for: The Metalloproteinase adam19b Is Required for Sensory Axon Guidance in the Hindbrain
Source: Front Neural Circuits. 2019 Mar 6;13:14. doi: 10.3389/fncir.2019.00014 (PMC6415755; doi:10.3389/fncir.2019.00014)
Supplement: TABLE S2 — Morpholino oligonucleotides. [file Table_2.docx]

Supplementary Table 2: Morpholino oligonucleotides

| ADAM19b Morpholino MO1 | TTAAATGCACGCTCCGAAGCATCGT |
| --- | --- |
| ADAM19b Morpholino MO2 | AACATGCAATTTGGACTTACCCCAT |
| ADAM19b Exon16.F | CCGGTCAACAGAAGATTCTGTGC |
| ADAM19b Exon18.R | GACCCGATGTCCCACAAAATGGT |
