# Supplementary material for: The Metalloproteinase adam19b Is Required for Sensory Axon Guidance in the Hindbrain
Source: Front Neural Circuits. 2019 Mar 6;13:14. doi: 10.3389/fncir.2019.00014 (PMC6415755; doi:10.3389/fncir.2019.00014)
Supplement: TABLE S3 — Primers used to produce a 600 bp PCR fragment for whole mount in situ hybridization. [file Table_3.docx]

Supplementary Table S3: Primers used to produce a 600 bp PCR fragment for whole mount *in situ* hybridization.

| Adam19insitu.F | EcoR1  TATAGAATTCATGCTTCGGAGCGTGCATTT |
| --- | --- |
| Adam19insitu.R | XhoI  TATACTCGAGCGGTTACTAGCTGAGCATTG |
