# Supplementary material for: The Metalloproteinase adam19b Is Required for Sensory Axon Guidance in the Hindbrain
Source: Front Neural Circuits. 2019 Mar 6;13:14. doi: 10.3389/fncir.2019.00014 (PMC6415755; doi:10.3389/fncir.2019.00014)
Supplement: TABLE S4 — Primers for Crispr/Cas9 targeting and PCR. [file Table_4.docx]

Supplementary Table 4: Primers for Crispr/Cas9 targeting and PCR

| Adam19b exon 2 target sequence | CAGACACAAAAGATCTGAGATGG |
| --- | --- |
| Adam 19b exon 2F PCR primer | TGTGTTGTGTGGAAGCAGTGTA |
| Adam 19b exon 2R PCR primer | TGGCGTATGACAATAAAAGCAC |
